# Cardiovascular magnetic resonance insights into anomalies of the mitral valve apparatus in Fabry cardiomyopathy and hypertrophic cardiomyopathy

**DOI:** 10.3389/fcvm.2024.1458705

**Published:** 2024-09-30

**Authors:** Lara Tondi, Giandomenico Disabato, Paolo D’Andria, Andrea Attanasio, Gianluigi Guida, Federico Pieruzzi, Giada De Angeli, Marco Canepa, Gianpaolo Carrafiello, Massimo Piepoli, Pietro Spagnolo, Massimo Lombardi, Antonia Camporeale

**Affiliations:** ^1^ Multimodality Cardiac Imaging Section, IRCCS Policlinico San Donato, Milan, Italy; ^2^ Postgraduate School in Radiodiagnostics, Università Degli Studi di Milano, Milan, Italy; ^3^ Cardiovascular Diseases, Cardiology Department, University of Milan, Milan, Italy; ^4^ Clinical Cardiology, IRCCS Policlinico San Donato, Milan, Italy; ^5^ Nephrology, Fondazione IRCCS San Gerardo dei Tintori, Monza, Italy; ^6^ Department of Medicine and Surgery, University of Milano-Bicocca, Milan, Italy; ^7^ Health Professions Research and Development Unit, IRCCS Policlinico San Donato, San Donato Milanese, Italy; ^8^ Cardiovascular Unit, IRCCS Ospedale Policlinico San Martino, Genova, Italy; ^9^ Department of Internal Medicine, University of Genova, Genova, Italy; ^10^ Department of Diagnostic and Interventional Radiology, Foundation IRCCS Cà Granda-Ospedale Maggiore Policlinico, Milan, Italy; ^11^ Department of Biomedical Sciences for Health, University of Milan, Milan, Italy

**Keywords:** cardiovascular magnetic resonance, hypertrophic cardiomyopathy, Fabry cardiomyopathy, mitral valve apparatus abnormalities, myocardial hypertrophy, papillary muscles

## Abstract

**Background and aims:**

Despite different etiopathogenesis, Fabry Disease cardiomyopathy (FDc) and sarcomeric hypertrophic cardiomyopathy (HCM) share a similar hypertrophic phenotype, including anomalies of the mitral valve apparatus (AMVA). Some of these anomalies have also been described in the pre-hypertrophic stage of both diseases. This cardiovascular magnetic resonance (CMR) study aimed to: (i) compare AMVA between FDc and HCM with a similar degree of left ventricular hypertrophy (LVH), to add new insights into differential diagnosis; (ii) assess whether AMVA represent an early and progressive alteration in FDc; (iii) propose simple and potentially reproducible measurements of AMVA.

**Methods:**

This observational, retrospective study enrolled: (i) 80 Fabry patients, divided into three groups with increasing severity of cardiac phenotype (20 patients LVH-/normal T1, 20 patients LVH-/low T1 and 40 patients LVH+), and (ii) 40 patients with HCM. All patients underwent CMR. The LVH + FDc and the HCM groups were matched for age, sex, body surface area and left ventricular (LV) mass. The following AMVA were measured on cine images: papillary muscles (PMs) hypertrophy (maximal diameter (Dmax) of anterolateral (Al) and posteromedial (Pm) PM), apical displacement, anteriorization of Al PM and anterior mitral valve leaflet (AMVL) elongation. Reference values for defining AMVA were derived from a matched healthy control group (*n* = 40).

**Results:**

Both HCM and FDc LVH + patients showed PMs hypertrophy, with a greater degree in the FDc LVH + group [Dmax Al PM 16 ± 3.4 vs. 15 ± 3.1 mm, *p* 0.017; Dmax Pm PM 14 ± 4.0 vs.12 mm (10.0–14.0), *p* 0.039] As compared to controls, both HCM and FDc LVH + patients showed PMs apical displacement (HCM 83% vs. healthy volunteers 8%, *p* < 0.001; FDc LVH + 65% vs. healthy volunteers 8%, *p* < 0.001), with a greater prevalence in HCM. Anteriorization of Al PM was only evident in HCM (15 ± 6.2 vs. healthy controls 21 ± 5.3 mm, *p* < 0.001). Elongation of AMVL was detected both in HCM and FDc with LVH + (HCM 29 ± 4.0 vs. healthy volunteers 24 ± 2.9 mm, *p* < 0.001; FDc LVH + 27 ± 4.0 vs. healthy volunteers 24 ± 2.9 mm, *p* < 0.001) without significant differences between the two phenocopies. The prevalence of myocardial crypts was higher among HCM patients than in FDc LVH + patients (75% vs. 48%, *p* 0.012).

**Conclusions:**

we report greater PMs hypertrophy in FDc and a higher prevalence of PMs positional alterations (anterior and apical displacement) and myocardial crypts in HCM. All these AMVA became more pronounced with the progression of the FDc phenotype. We suggest the systematic inclusion of the analysis of AMVA by simple linear measurements on cine images in the CMR assessment of hypertrophic cardiomyopathies, to help in the differential diagnosis between HCM and FDc and to facilitate early detection of cardiac involvement in FDc.

## Graphical Abstract

The figure presents the study's main findings, focused on the characterization of AMVA. The study proposes simple and reproducible measurements of mitral valve apparatus parameters on routinely acquired cine SSFP sequences. The central box in grey displays normal values of these measurements, derived from the cohort of healthy controls. Patients with HCM showed a prevalence of PMs positional abnormalities, while in FDc patients PMs hypertrophy prevailed, and AMVA enhanced with the progression of the disease phenotype. Al PM, anterolateral papillary muscle; AMVL, anterior mitral valve leaflet; cineSSFP, cine steady-state free precession; Dmax, maximum diameter; FDc, Fabry cardiomyopathy; FD, Fabry disease; HCM, hypertrophic cardiomyopathy; LVH, left ventricular hypertrophy; Pm PM, posteromedial papillary muscle.

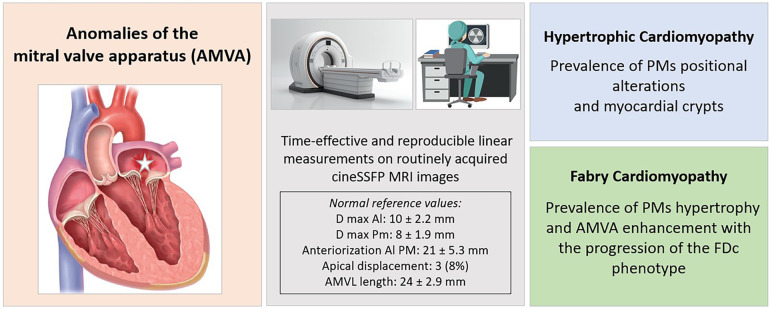

## Background

Anomalies of the mitral valve apparatus (AMVA), including hypertrophy of the papillary muscles (PMs), anterior displacement, apical displacement, and elongation of the anterior mitral valve leaflet (AMVL), have frequently been observed in association with left ventricular hypertrophy (LVH), as seen in Anderson-Fabry cardiomyopathy (FDc) (1) and hypertrophic cardiomyopathy (HCM) (2–6). These two phenocopies stem from different pathophysiological backgrounds. In FDc, LVH is triggered by the intracellular lysosomal accumulation of glycosphingolipids (globotriaosylceramide, Gb3) in all cardiac cell types, due to a partial or total deficiency in alpha-galactosidase A enzyme activity. Conversely, in HCM, primary dysfunction of the sarcomere leads to impaired excitation-contraction coupling (7), resulting in cardiomyocyte disarray and LVH.

In FDc, PMs exhibit disproportionate hypertrophy (8). This feature is more pronounced in FD compared to other hypertrophic phenotypes (9) and can be detected even in the absence of LVH (10, 11).

Cardiac Magnetic Resonance (CMR) plays a pivotal role in assessing cardiomyopathies, as it combines volumetric and functional evaluation with tissue characterization, and provides good spatial resolution to define the morphology of small structures such as PMs and mitral leaflets. However, current literature lacks standardization of measurements and normality ranges for the evaluation of the mitral valve apparatus, making its characterization arbitrary.

The aims of the present study are to: (i) compare AMVA between FDc and HCM with a similar degree of LVH, which may be useful for differential diagnosis between the two phenocopies; (ii) assess whether AMVA represent an early and progressive alteration in the cardiomyopathic spectrum of FDc; (iii) propose simple and potentially reproducible linear measurements of AMVA, applicable to commonly acquired CMR cine steady-state free precession (cineSSFP) sequences, in a time-saving manner.

## Methods

### Study population

This single-centre, observational and retrospective study enrolled: (i) 80 patients with genetically confirmed Fabry disease, divided into three groups according to the increasing severity of cardiac phenotype (20 patients LVH-/normal T1, 20 patients LVH-/low T1 and 40 patients LVH+), and (ii) 40 patients with HCM. The study includes only FD patients with pathogenetic or likely pathogenetic variants, while variants of unknown significance (VUS) have been excluded. All patients were referred for CMR to the Multimodality Cardiac Imaging Unit of IRCCS Policlinico San Donato (San Donato Milanese, Milan Italy), from 2016 to 2023.

In FD, LVH was defined as a maximal wall thickness (MWT) ≥ 13 mm in one or more left ventricular (LV) myocardial segments (12, 13) and/or increased LV mass index (14). In HCM LVH was defined according to the 2023 Guidelines of the European Society of Cardiology (15), as a MWT ≥ 15 mm which cannot be explained by abnormal loading conditions, or ≥13 mm in consideration of other features including family history, genetic findings, and electrocardiographic abnormalities.

The LVH + FDc and the HCM groups were matched for age, sex, body surface area (BSA) and LV mass. Defining subgroups based on LVH would be arbitrary since there are no specific cut-off values of LV mass or MWT for grading LVH. Given the extremely heterogeneous LVH pattern exhibited by HCM and FD patients, we choose to match them for LV mass, rather than for MWT, since myocardial mass might better reflect the progression of LVH compared to an isolated and regional MWT value, which has also proven to be a poorly reproducible parameter (16). Since in our FDc cohort no patients exhibited isolated apical LVH, HCM patients with apical phenotype were excluded, in order to identify the prevalence and type of AMVA between phenocopies with comparable LVH patterns. Of note, a higher prevalence of apical PMs displacement has been recently reported in apical HCM (17). Other exclusion criteria were prior surgical myectomy/alcohol septal ablation, age <18 years, unwillingness/inability to provide informed consent, any contraindication to CMR and poor image quality.

Patients were compared to a group of 40 gender, age and BSA-matched controls, sourced from a local database of healthy volunteers, without cardiovascular disease or significant comorbidities, with non-pathological electrocardiogram and CMR parameters within normality ranges (14).

The research protocol was approved by the local Ethics Committee (Protocol identification number 109/int/2019) and complied with the Declaration of Helsinki. Informed consent was obtained from all participants.

### CMR protocol and image analysis

CMR was performed on a 1.5 T magnet (MAGNETOM Aera, Siemens Healthcare, Erlangen Germany). Each scan included: (i) scout images (ii) balanced cineSSFP images in LV short-axis and at least 3 long-axis views. Sequence parameters were slice thickness, 8.0 mm; no gap; flip angle, 60°−80°; repetition time, 3.8 ms; echo time, 1.7 ms; typical readout field of view, 350 mm; phase resolution matrix, 75%; voxel size 1.4 × 1.4 mm; mean temporal resolution ∼33 ms; (iii) Shortened Modified Look-Locker inversion recovery sequence (ShMOLLI; Work-in-Progress # 780B VD13A-SP4; basal, mid-ventricular and apical short axis, 3 long-axis views,) before and 15 min after 0.1 mmol/kg of contrast (Gadovist, Bayer Schering Pharma, Berlin, Germany) for T1 mapping; (iv) two-dimensional gradient echo inversion recovery LGE images in LV short-axis and long-axis views, acquired 8–10 min after contrast administration.

LV volumes, mass and ejection fraction (EF) were calculated from cineSSFP images and indexed to BSA using the thresholding method on a commercially available software (Qmass, MR version 6.2.1; Medis Medical Imaging Systems, Leiden, The Netherlands). Both PMs and trabeculae were included in the computation of global LV mass. According to the AHA 16 segments model the MWT was measured in cineSSFP images for each myocardial segment. Late gadolinium enhancement (LGE) was quantified as % of LV mass using the standard deviations (SDs) method with a 5 SDs cut-off. Inline-generated T1 maps were analyzed using Argus software (Siemens Healthcare, Erlangen, Germany). In HCM patients two regions of interest (ROI) were manually drawn in two sites of LGE-negative myocardium, on pre- and post-contrast maps, favouring the basal and mid-interventricular septum; pre and post-contrast T1 values were obtained by averaging the two ROIs measurements. In FDc patients two ROIs were systematically drawn, one in the basal interventricular septum and the other in the basal infero-lateral wall, the latter being the usual location of fibrosis/inflammation. Extracellular volume (ECV) of remote myocardium (r-ECV) was calculated as ECV = (1-Hct)[*Δ*R1 myocardium]/[*Δ*R1 blood]. Blood samples for hematocrit were obtained at the time of CMR. Site-specific upper reference limits were 956 ± 34 ms for native T1 and 27 ± 2% for ECV, measured on ShMOLLI sequences.

### Evaluation of the anomalies of the mitral valve apparatus

**Figure 1:**
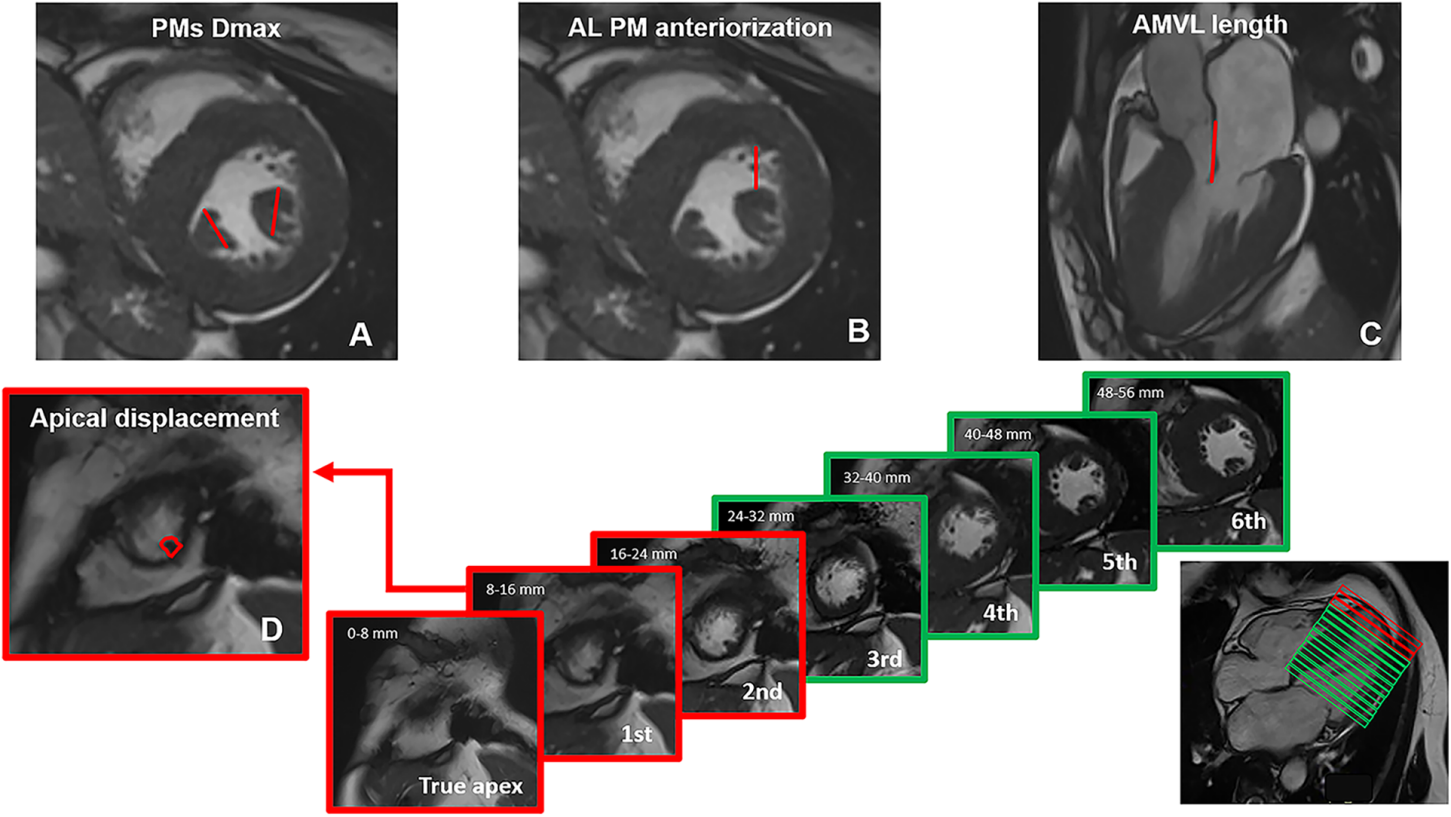
Methods used for measuring mitral valvular apparatus anomalies on CMR cine images. **(A)** papillary muscles (PMs) hypertrophy was expressed as the maximum diameter (Dmax) of anterolateral (Al) and posteromedial (Pm) PMs on short-axis images; **(B)** anteriorization of Al PM was described as the distance in mm between the Al PM and the anterior interventricular junction; **(C)** anterior mitral valve leaflet (AMVL) length measurement was performed in 3-chamber images, with maximally extended leaflets parallel to the anterior septum and LV free wall during diastole; **(D)** apical displacement was classified by counting the number of short axis slices, starting from the apex, where the first distal insertion of the PM appeared; based on the healthy control cohort, distal PMs insertion was considered normal when it could be detected in or above the third slice. All measurements were acquired in diastole.

The methods used for measuring AMVA are depicted in Figure 1.

PMs measurements were performed in diastole on short-axis cineSSFP images, and included the following parameters: (i) PMs hypertrophy was expressed as the maximum diameter (Dmax) of antero-lateral (Al) and postero-medial (Pm) PMs; (ii) apical displacement was classified by counting the number of slices, starting from the apex, where the first distal insertion of the PM appeared; (iii) anteriorization of Al PM was described as the distance in mm between the Al PM and the anterior interventricular junction.

AMVL length measurement was performed in diastole, in 3-chamber cineSFFP images, with maximally extended leaflets parallel to the anterior septum and LV free wall (4). Myocardial crypts were defined as narrow, deep blood-filled invaginations penetrating >50% of the thickness of the adjoining myocardium during diastole (6).

Due to the lack of normality ranges for AMVA, the matched healthy control group was used as the reference.

Two expert operators (L.T. and A.C., Level 3 EACVI CMR Certification), blinded to clinical data, analyzed CMR images and performed intra and inter-observer reproducibility analysis for mitral valve apparatus anomalies on a subset of 30 randomly chosen patients. For intra-observer variability the same operator reanalyzed the same data set weeks apart and blinded from the first measurements; for inter-observer variability, the second operator independently and blindly analyzed the same images.

### Statistical analysis

Statistical analyses were performed using SPSS version 24.0 (IBM SPSS, IBM Corporation, Armonk, NY, USA). Categorical variables were shown as count (n) and percentage (%) and compared with the *χ*2 test. The Kolmogorov–Smirnov test was used to assess the normal distribution of data collected. Normally distributed variables were expressed as mean (M) ± standard deviation (SD) and compared with the T student test and analysis of variance; non-normally distributed variables were expressed as median and interquartile range and compared with Mann Whitney *U*-test and non-parametric analysis of variance (Kruskal Wallis test).

Intra- and inter-observer reproducibility of mitral valve apparatus anomalies measurements were assessed using Bland–Altman plots and intraclass correlation coefficients (ICCs) for continuous variables, and Coehn's K coefficient for dicotomic variables. Significance was defined as a *P*-value < 0.05.

## Results

### Study population

The study population included 160 subjects: 40 healthy volunteers (age 53 ± 11.8, 67% males), 40 patients with FDc LVH + (age 54 ± 10.1, 67% males), 20 patients with FDc LVH-/low T1 (age 32 ± 10.1, 70% males), 20 patients with genetic diagnosis of FD and no CMR signs of cardiac involvement (LVH-/normal T1, age 28 ± 13.1, 25% males) and 40 patients with HCM (age 54 ± 14.8, 67% males).

Supranormal LV EF was the only distinguishing parameter between healthy controls and Fabry patients without signs of cardiac involvement (FD LVH-/normal T1 70 ± 6.5% vs. healthy volunteers 66 ± 7.1%, *p* < 0.001). Our data confirm that progressive myocardial involvement in FD encompasses the lowering of native T1, the development of LVH with increasing LV mass index and the appearance of LGE.

Patients with hypertrophic FDc showed lower native septal T1 and ECV (native T1: 848 ± 50.9 ms vs. 970 ± 34.4 ms, *p* < 0.001; ECV: 26 ± 2.5% vs. 29 ± 3.3%, *p* < 0.001) than HCM patients. The prevalence of myocardial crypts was higher among HCM patients than in FDc LVH + patients (75% vs. 48%, *p* 0.012).

Reference values for defining AMVA derive from the healthy volunteers’ cohort. Regarding apical displacement, we observed that in the healthy controls the distal insertion of the PMs typically appeared in or above the third slice. For reference, we counted the true apex as slice 0, which is defined as the slice cutting the apex without the left ventricular (LV) cavity visible in diastole. Since there is no gap between slices and each one is 8 mm thick, we defined apical displacement as the appearance of the distal insertion of PMs between 0 and 16 mm from the apex (i.e., 2 slices) (Figure 1). Baseline characteristics of the study populations are detailed in Table 1.

**Table 1:** Characteristics of the study population.

	Healty volunteers (40)	FDc LVH+ (40)	FDc LVH-Low T1 (20)	FD LVH-Normal T1 (20)	HCM (40)	*p*-Value
Demographic data						
Age (years)	53 ± 11.8^d^	54 ± 10.1	32 ± 10.1^b^	28 ± 13.1	54 ± 14.8	<0.001*
Male, *n* (%)	27 (67)	27 (67)	14 (70)	5 (25)	27 (67)	0.080
BSA (m^2^)	1.9 ± 0.1^d^	1.8 ± 0.3	1.8 ± 0.2	1.6 (1.6–1.8)^c^	1.9 ± 0.2	0.001*
CMR data						
LVEF (%)	66 ± 7.1^d^	72 ± 7.5	70 ± 5.4	70 ± 6.5	73 ± 8.7	<0.001*
LVEDVi (ml/m^2^)	73 ± 11.9	69 ± 14.8^a^	80 ± 12.9^b^	73 ± 11.4	62 (53.3–73.5)	<0.001*
LVESVi (ml/m^2^)	25 ± 6.5	18 (14.3–24.7)	24 ± 6.5^b^	22 ± 6.2	17 ± 7.9	<0.001*
SV (ml)	91 ± 20.3	89 (70–108)	98 ± 17.5	86 ± 11.9^c^	83 (72–96)	0.079
LV Mass i (g/m^2^)	60 ± 11.6	136 ± 40.4	70 ± 5.4^b^	56 ± 9.3^c^	127 (104.3–161.9)	<0.001*
LV MWT (mm)	–	17 (14.3–21.0)^a^	10 ± 1.3^b^	7 (7.0–8.0)^c^	20 (17.0–21.8)	<0.001*
Crypts, *n* (%)	8 (20)	19 (47%)^a^	3 (15%)^b^	2 (10%)	30 (75)	<0.001*
LGE (% LVmass)	0	4 (1.3–8.1)	0^b^	0	6 (2.0–13.0)	<0.001*
Septal T1 (ms)	–	848 ± 50.9^a^	872 ± 45.3	969 ± 25.6^c^	970 ± 34.4	<0.001*
Remote ECV (%)	–	26 ± 2.5^a^	26 ± 2.7	28 ± 2.2^c^	29 ± 3.3	<0.001*

Data are presented as mean ± SD, median and interquartile range or *n* (%).

BSA, body surface area; ECV, extracellular volume; FDc, Fabry cardiomyopathy; FD, Fabry disease; HCM, hypertrophic cardiomyopathy; LGE, late gadolinium enhancement; LV, left ventricular; LVEDV i, left ventricular end-diastolic volume index to BSA; LVEF, left ventricular ejection fraction; LVESV i, left ventricular end-systolic volume index to BSA; LVH, left ventricular hypertrophy; LV Mass i, left ventricular mass indexed to BSA; LVWT, maximum left ventricular wall thickness; SV, stroke volume.

^a^ *p* < 0.05 FDc LVH + vs. HCM

^b^ *p* < 0.05 FDc LVH + vs. FDc LVH-/lowT1

^c^ *p* < 0.05 FDc LVH-/low T1 vs. FDc LVH-/normal T1

^d^ *p* < 0.05 FDc LVH-/normal T1 vs. healthy controls.

* *P* < 0.05 considered statistically significant.

### Anomalies of the mitral valve apparatus in Fabry cardiomyopathy and hypertrophic cardiomyopathy

Both HCM and FDc LVH + patients showed PMs hypertrophy (Dmax Al PM HCM 15 ± 3.1 vs. healthy controls 10 ± 2.2 mm, *p* < 0.001; Dmax Pm PM HCM 12 (10.0–14.0) vs. healthy controls 8 ± 1.9 mm, *p* < 0.001; Dmax Al PM FDc LVH + 16 ± 3.4 vs. healthy controls 10 ± 2.2 mm, *p* < 0.001; Dmax Pm PM FDc LVH + 14 ± 4.0 vs. healthy controls 8 ± 1.9 mm, *p* < 0.001). Of note, in the FDc LVH + group, a greater degree of PMs hypertrophy was observed compared to HCM (Dmax Al PM 16 ± 3.4 vs. 15 ± 3.1 mm, *p* 0.017; Dmax Pm PM 14 ± 4.0 vs. 12 mm (10.0–14.0), *p* 0.039). As compared to controls, both HCM and FDc LVH + patients showed PMs apical displacement (HCM 83% vs. healthy volunteers 8%, *p* < 0.001; FDc LVH + 65% vs. healthy volunteers 8%, *p* < 0.001). Comparing the two phenocopies, the prevalence of PMs apical displacement was higher in the HCM cohort, although it did not reach statistical significance (*p* 0.075).

Anteriorization of Al PM was only evident in HCM (15 ± 6.2 vs. healthy controls 21 ± 5.3 mm, *p* < 0.001) and a significant difference was observed between the two phenocopies (HCM 15 ± 6.2 vs. FDc LVH + 18 mm (15.0–21.8), *p* 0.020).

Elongation of AMVL was detected both in HCM and FDc with LVH + (HCM 29 ± 4.0 vs. healthy volunteers 24 ± 2.9 mm, *p* < 0.001; FDc LVH + 27 ± 4.0 vs. healthy volunteers 24 ± 2.9 mm, *p* < 0.001); no significant differences were observed between the two phenocopies (27 ± 4.0 vs. 29 ± 4.0, *p* 0.078).

**Figure 2:**
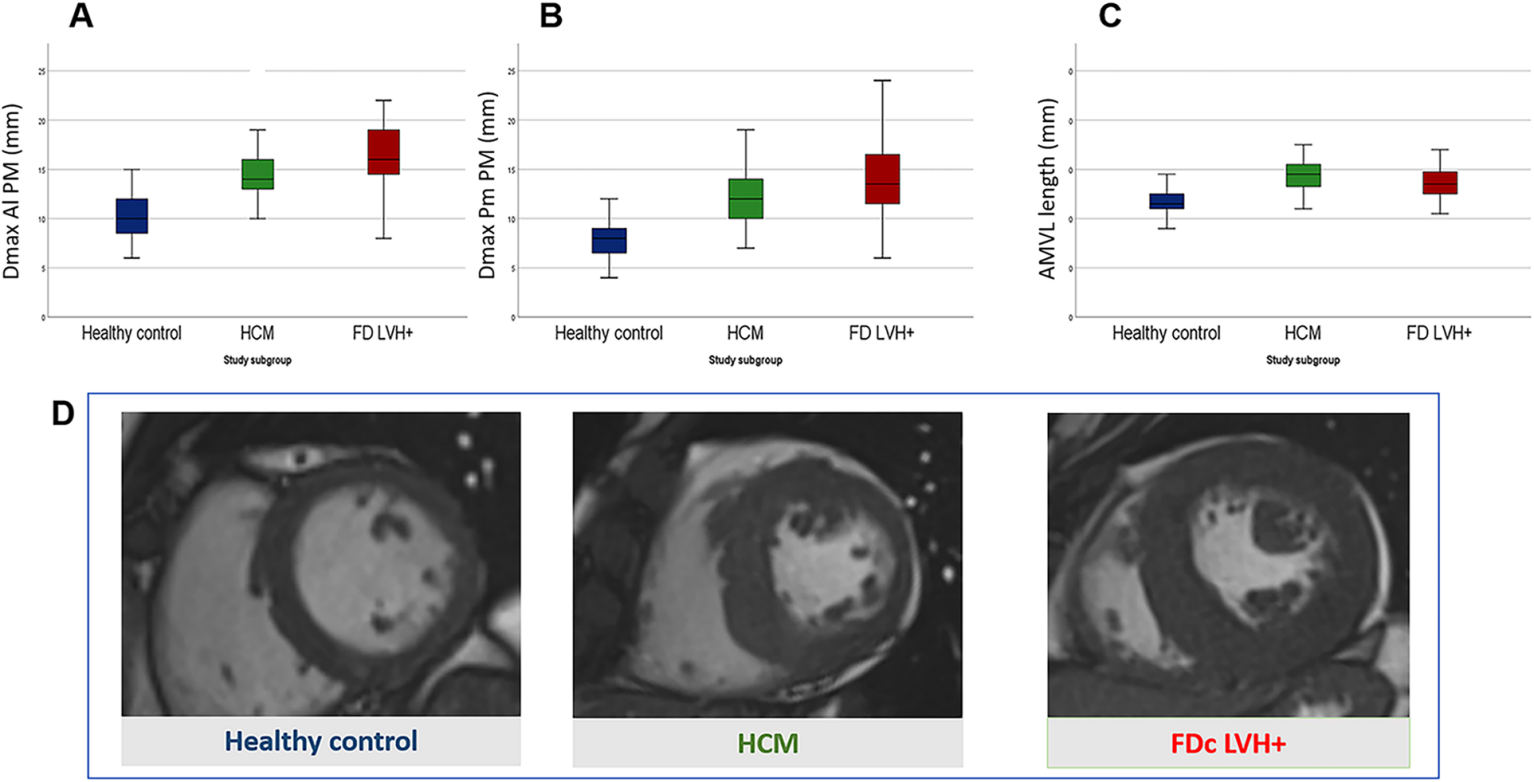
Comparison of mitral valve apparatus anomalies between Fabry disease cardiomyopathy (FDc) and hypertrophic cardiomyopathy (HCM) with similar degree of LVH. Box plots in **(A–C)** show measurements of the mitral valve apparatus in healthy controls (blue), HCM (green) and FDc (red) patients. Measurements are expressed in millimetres and are respectively in **(A)** the maximal diameter of posteromedial papillary muscle (Dmax Pm PM), in **(B)** the maximal diameter of anterolateral papillary muscle (Dmax Al PM) and in **(C)** the length of the anterior mitral leaflet (AMVL length). **(D)** Short axis cineSSFP images in diastole of a healthy control (blue), HCM (green) and FDc LVH + (red) patients.

All results are reported in Table 2 and illustrated in Figure 2.

**Table 2:** Comparison of mitral valve apparatus anomalies in Fabry cardiomyopathy and hypertrophic cardiomyopathy.

	Healty volunteers (40)	FDc LVH+ (40)	HCM (40)	Overall *p*-Value
Dmax Al PM (mm)	10 ± 2.2^b,c^	16 ± 3.4^a^	15 ± 3.1	<0.001*
Dmax Pm PM (mm)	8 ± 1.9^b,c^	14 ± 4.0^a^	12 (10.0–14.0)	<0.001*
Anteriorization of Al PM (mm)	21 ± 5.3^c^	18 (15.0–21.8)^a^	15 ± 6.2	<0.001*
PM apical displacement *n*, (%)	3 (8)^b,c^	26 (65)	33 (82)	<0.001*
AMVL length (mm)	24 ± 2.9^b,c^	27 ± 4.0	29 ± 4.0	<0.001*

Data are presented as mean ± SD, median and interquartile range or *n* (%).

Al PM, antero-lateral papillary muscle; AMVL, anterior mitral valve leaflet; Dmax, maximum diameter; FDc, Fabry cardiomyopathy; HCM, hypertrophic cardiomyopathy; LVH, left ventricular hypertrophy; Pm PM, postero-medial papillary muscle.

^a^ FDc LVH + vs. HCM.

^b^ FDc LVH + vs. healthy controls.

^c^ HCM vs. healthy controls.

* *P* < 0.05 considered statistically significant.

### Anomalies of the mitral valve apparatus across Fabry cardiomyopathy

**Figure 3:**
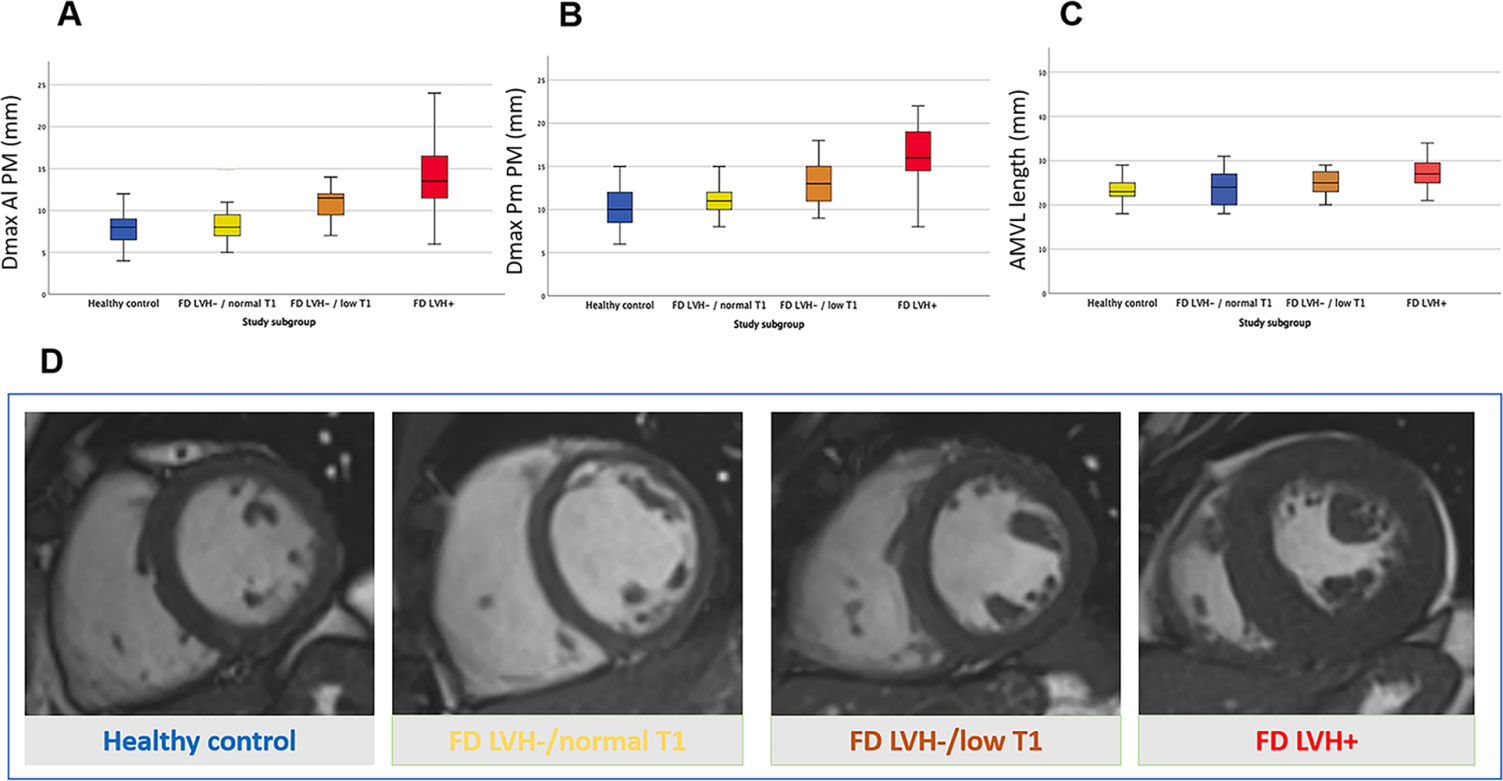
Anomalies of the mitral valve apparatus across Fabry cardiomyopathy: Fabry disease without left ventricular hypertrophy and normal myocardial T1 (FD LVH-/normal T1), Fabry disease without left ventricular hypertrophy and with low myocardial T1 suggestive for cardiac storage disease (FD LVH-/lowT1) and Fabry disease with cardiac hypertrophy (FD LVH+). Box plots in **(A–C)** show the progressive increase of mitral valve apparatus measurements alongside the worsening of FD cardiomyopathy; the measurements are expressed in millimetres and are respectively in **(A)** the maximal diameter of posteromedial papillary muscle (Dmax Pm PM), in **(B)** the maximal diameter of anterolateral papillary muscle (Dmax Al PM) and in **(C)** the length of the anterior mitral leaflet (AMVL length). **(D)** Short axis cineSSFP images in diastole of a healthy control (blue), FD LVH-/normal T1 (yellow), FD LVH-/reducedT1 (orange) and FD LVH + (red) patients.

With the increasing severity of cardiac involvement across FD groups, we observed a parallel progression in the magnitude of AMVA (i.e., Dmax Al PM, Dmax Pm PM, PMs apical displacement and AMVL length) (Figure 3). No significant differences were observed between healthy volunteers and FD patients without detectable storage, although a trend towards an increased prevalence of apical displacement was observed (8% vs. 25%, *p*. 0.060). The first alterations in mitral valve apparatus emerge in FDc LVH-/low T1 and become overt in the FD LVH + cohort. Of note, PMs hypertrophy and apical displacement are appreciable in pre-hypertrophic FD patients with the lowering of native T1 (FDc LVH-/lowT1 vs. healthy controls: Dmax Al PM 13 ± 2.8 vs. 10 ± 2.2, *p* < 0.001, Dmax Pm PM 11 ± 2 vs. 8 ± 1.9, *p* < 0.001, apical displacement 45 vs. 8%, *p* < 0.001). All results are detailed in Table 3.

**Table 3:** Anomalies of the mitral valve apparatus across Fabry cardiomyopathy.

	Healty volunteers (40)	FD LVH- Normal T1 (20)	FDc LVH-Low T1 (20)	FDc LVH+ (40)	Overall *p* Value
Dmax Al PM (mm)	10 ± 2.2	11 (10.0–12.0)	13 ± 2.8^b,c^	16 ± 3.4^a^	<0.001*
Dmax Pm PM (mm)	8 ± 1.9	8 (7.0–9.8)	11 ± 2.0^b,c^	14 ± 4.0^a^	<0.001*
Anteriorization of AL PM (mm)	21 ± 5.3	19 ± 3.2	20 (16.0–22.0)	18 (15.0–21.8)	0.237
PM apical displacement *n*, (%)	3 (8)	5 (25)	9 (45)^c^	26 (65)	<0.001*
AMVL length (mm)	24 ± 2.9	24 ± 3.9	25 ± 2.8	27 ± 4.0^a^	<0.001*

Data are presented as mean ± SD, median and interquartile range or *n* (%).

Al PM, antero-lateral papillary muscle; AMVL, anterior mitral valve leaflet; Dmax, maximum diameter; FDc, Fabry cardiomyopathy; FD, Fabry disease; LVH, left ventricular hypertrophy; Pm PM, postero-medial papillary muscle.

^a^ FDc LVH + vs. FDc LVH-/lowT1.

^b^ FDc LVH-/low T1 vs. FDc LVH-/normal T1.

^c^ FDc LVH-/low T1 vs. healthy controls.

* *P* < 0.05 considered statistically significant.

### Intra and interobserver reproducibility of AMVA measurements on cine images

Intra ed interobserver reproducibility of AMVA measurements on cine images (Dmax PMs, AMVL length and anteriorization of Al PM) was good to excellent, with small biases and ICC ranging from 0.89 to 0.98. Intra and interobserver reproducibility results for AMVA measurements are reported in Table 1 of Supplementary Files.

## Discussion

The main findings of the present study are: (i) in the presence of LVH of a similar degree, PMs hypertrophy is more pronounced in the FDc group, whereas PMs positional alterations (anterior and apical displacement), and myocardial crypts, are more evident in the HCM group; (ii) PMs hypertrophy and apical displacement, AMVL elongation and myocardial crypts become increasingly apparent with the progression of the FDc phenotype; (iii) the proposed method for assessing AMVA is time-effective and demonstrates good intra- and inter-observer variability. The main findings are presented in the Graphical abstract.

AMVA have been reported both in HCM and in FDc, likely due to secondary common pathways activated by a different initial trigger represented by abnormal sarcomere protein function and glycosphingolipid storage, respectively. In FDc, disproportionate hypertrophy of the PMs has been described (8, 9), exceeding that seen in HCM and significantly affecting the estimation of LV mass. Even in patients with HCM the PMs are frequently hypertrophied, and the hypertrophy of PMs correlates with LV wall thickness and myocardial mass (18). The distance between the PMs and the septum and PMs apical displacement are also points of interest in patients with HCM, with a potential impact on dynamic outflow tract obstruction (19). Elongation of AMVL was first observed in HCM as a part of a subclinical cardiac phenotype, alongside the presence of myocardial crypts (20). Following studies demonstrated the same abnormalities in FDc (21, 22).

To our knowledge, this is the first CMR study to evaluate AMVA with a head-to-head comparison between FDc and HCM with similar degrees of LVH. We confirmed that FDc LVH+ patients exhibit greater PMs hypertrophy (9). On the other hand, HCM patients display more evident PMs positional abnormalities (apical displacement and anteriorization of Al PM). Finally, in comparing the two phenocopies, the prevalence of myocardial crypts is significantly higher in HCM patients than in FDc, where it is known as an early marker of disease (23). The different alterations in AMVA observed in the two phenocopies could be interpreted based on their different etiopathologies. As a storage disease, in FD, the glycosphingolipid accumulation could explain the predominant and progressive hypertrophy of PMs. Conversely, despite the two cohorts sharing comparable LV mass, HCM patients more frequently show asymmetry of the parietal hypertrophy, associated with greater anatomical distortion of the left ventricle, and this could explain the higher prevalence and extent of positional anomalies of the PMs.

It must be acknowledged that differences in AMVA have a limited impact on the differential diagnosis between HCM and FDc when considered in isolation, and native T1 remains the cornerstone for this purpose (24). However, given the drastic implications of differential diagnosis on clinical management, we strongly support the integration of all the information deriving from CMR in a multiparametric evaluation to differentiate between FDc and HCM with similar degrees of LVH (25).

In the field of FD, detailing AMVA may also be useful for the early detection of heart damage, which is the main driver of prognosis (26, 27). Prompt identification of cardiac involvement in FD is a major challenge, entailing relevant therapeutic and prognostic implications (21, 28). In this regard, our group has already described early-onset morpho-functional and electrocardiographic alterations (29–31), showing progressive enhancement throughout the spectrum of FDc. PMs hypertrophy has been previously reported to precede overt LVH in patients with FDc, as demonstrated by echocardiography in a population of FD LVH- patients (32). This study, however, did not include the evaluation of native T1 to distinguish between the presence or absence of detectable storage. Interestingly, when subtyping the FD LVH- population, we confirmed that PMs hypertrophy is already present in LVH-/low T1 patients, suggesting it as an early morphological marker of the disease. On the other hand, PMs hypertrophy was not evident in patients without non-invasively detectable myocardial storage (LVH-/normal T1). Previous findings in this latter population are discordant. Kozor et al. (8) observed the presence of PMs hypertrophy, expressed as % of LV mass, even in LVH-/normal T1 patients, while Nordin et al. (11) found no significant differences in PMs mass between healthy volunteers and FD patients without detectable storage. Indeed, in our population, we noted an increasing trend in the diameter of the anterior Al PM, although it did not reach statistical significance. Across FDc groups, we also observed a trend towards a progressive increase in the prevalence of PM apical displacement, myocardial crypts and AMVL elongation. Overall, the concept that in FDc mitral valve apparatus anomalies advance with the progression of damage remains consistent across all studies; discrepancies in results may depend on different methodologies used for measurement. This study confirms that in FDc women exhibit a lesser degree of LVH compared to men. Having shown that valvular apparatus abnormalities follow the structural evolution of the disease, we expect that these abnormalities might be less evident in women as well. Of note, despite the lesser degree of hypertrophy in women, it has been shown that this does not imply a better cardiovascular prognostic profile (33).

Finally, while reference standards exist for tissue parameters and major morpho-structural indices, AMVA are usually reported descriptively, hence arbitrarily, in daily practice. There is no standardization for quantification nor ranges of normal values. Indeed, various studies in the literature report different methodologies (9, 10, 17, 32, 34) for AMVA analysis. This work proposes a simple, reproducible and time-effective method that allows the evaluation of these parameters, using linear measurements on cineSSFP images, which are routinely acquired in any CMR examination.

In conclusion, when comparing FDc and HCM, we report greater PMs hypertrophy in FDc and a higher prevalence of PMs positional alterations (anterior and apical displacement) and myocardial crypts in HCM. All these anomalies become more pronounced with the progression of the FDc phenotype. We suggest the systematic inclusion of the analysis of AMVA in the CMR assessment of cardiomyopathies with a hypertrophic phenotype using simple and reproducible linear measurement on cineSSFP images. This approach not only aids in the differential diagnosis between HCM and FDc but also facilitates the early detection of cardiac involvement in FD, thereby definitively impacting clinical and therapeutic management.

### Limitations

In addition to the single-centre and retrospective design of the study, the following limitations must be mentioned. The exclusion of cases with pure apical HCM from the analysis: (i) prevents extending the current findings to this subgroup of patients and (ii) likely led to an underestimation of the real prevalence of PM anomalies in the HCM group, known to be more frequent in the apical phenotype (17). Given the sample size, a specific gender-based analysis within the Fabry population was not achievable. Information about genetic testing in the HCM cohort is not available for all patients. The lack of longitudinal follow-up hinders the interpretation of the prognostic impact of AMVA in FDc and HCM.

## Data Availability

The raw data supporting the conclusions of this article will be made available by the authors, without undue reservation.
