# Supplementary material for: Cardiovascular magnetic resonance insights into anomalies of the mitral valve apparatus in Fabry cardiomyopathy and hypertrophic cardiomyopathy
Source: Front Cardiovasc Med. 2024 Sep 30;11:1458705. doi: 10.3389/fcvm.2024.1458705 (PMC11475249; doi:10.3389/fcvm.2024.1458705)
Supplement: Supplementary file 1 [file Table1.pdf]

Table 1S: Reproducibility analysis for papillary muscle anomalies measurements

**Intra-observer reproducibility**	**Bland Altman analysis (bias, LOA)**	**ICC (95% CI)**
Dmax Al PM (mm)	0.20 (4.36/-3.96)	0.97(0.94-0.99)
Dmax Pm PM (mm)	-0.23 (3.13/-3.60)	0.97 (0.94-0.99)
Anteriorization of Al PM (mm)	-0.40 (4.47/-5.27)	0.98 (0.95-0.99)
AMVL length (mm)	1.6 (6.74/-3.54)	0.91 (0.74-0.96)
**Inter-observer reproducibility**	**Bland Altman analysis (bias, LOA)**	**ICC (95% CI)**
Dmax Al PM (mm)	0.58 (5.13/-4.46)	0.95(0.92-0.98)
Dmax Pm PM (mm)	0.52 (4.98/-4.17)	0.95 (0.93-0.98)
Anteriorization of AL PM (mm)	0.7 (4.90/-4.48)	0.94 (0.88-0.97)
AMVL length (mm)	1.9 (6.92/-4.35)	0.89 (0.73-0.94)

Al PM, antero-lateral papillary muscle; AMVL, anterior mitral valve leaflet; CI, confidence interval; Dmax, maximum diameter; ICC, intraclass correlation coefficient, LOA, limits of agreement; Pm PM, postero-medial papillary muscle.
